# Strange Phenomena in the Human Teeth, Etc.

**Published:** 1862-09

**Authors:** W. H. Shadoan


### STRANGE PHENOMENA IN THE HUMAN TEETH, ETC.

BY W. H. SHADOAN.

THAT there are strange developments in the human teeth, no one will pretend to deny. Then the object of this paper will be to give a few cases that have come under the observation of the author,—at the same time stating, that owing to the peculiarity of some of them, they may be doubted. But I can assure such as disbelieve that they are true as I shall give them.

I remember that in the year 1840 or 1841, in the town of S., Ky., there lived an old Revolutionary soldier. I had been acquainted with him ever since my infancy, and of course was taught to reverence him above other men of like age and standing, from the fact of his being one of the "god fathers" of the Republic. Well, as I stated above, this old man, in the time before stated, commenced erupting *a third set of teeth.* This excited community considerably, and of course every body knew it. In about three or four weeks the old hero had as well developed set of teeth as at twenty! The physicians were consulted, but could tell nothing about the cause. And what was more strange still, his eye-sight, which had failed him several years, was restored, so that he could see to read without spectacles. Almost immediately after this strange occurrence, the old man sickened and died. I have heard of several instances of the partial eruption of the teeth, but never of the whole denture, except this case. Though singular as it may seem, it is true, as I recollect it. I was living there at the time.

In the same county it was reported (but as to the truth of the statement I can not vouch) that a woman by the name of Weddle gave birth to a child who had several teeth well developed, and that the arch was completed in a few weeks after birth, and that it had considerable beard in a few weeks also. The child died. That there were some teeth in the child above named, I do not doubt. I heard several persons, who said they knew, say that the child had teeth and beard.

Theodore Hill, of B., Ohio, gave me the circumstances of the death of one of his children, which died in the National Hotel, Louisville, Ky., last March. He said the child was taken sick while on the way from Nashville, and when he got to the hotel, he sent for one of the best physicians (as was recommended) in the city, and the physician told him the child would die; that it was erupting all the teeth at once, and that the inflammation and pain were too great for a child of its age.

In the summer of 1857, a young man called on me to have some teeth filled and a few replaced. On examination, I found the lower jaw containing three incisors, one central and two lateral, one lateral in superior jaw, the other two were lost. On first examination, I concluded that he had lost one out of the lower and one out of the upper, and had forgotten it; but he stood out so stoutly he had not, and from the looks of the parts, I was convinced that the three incisors in each jaw were all that he had ever erupted.

In the following winter a young man of 18 years of age called to have the superior cuspids extracted. They had been forced out of line by the other teeth being so crowded that there was not room for them. I examined the teeth, and found to remove them would not only remove the deformity, but leave a nice full arch beneath. He had six incisors, two centrals, and four laterals—two on each side of the central incisors. One case had three, and the other six, neither one of them right.

March 6, 1862.—Two boys called to have teeth extracted.

One had a supernumerary tooth situated between the central incisors ; the other had two supernumerary teeth, one between the central incisors, and the other between the left central and left lateral incisor. Supernumerary teeth are not so unfrequently met with as a few years ago; but to have two patients in one day, and they from the same neighborhood, is very uncommon.

On the 3d of July, 1862, J. D., a gentleman of about 40 years of age, while in a conversation on the subject of dentistry, remarked that he had the most singular tooth I ever saw, and asked me to look at it. The right superior cuspidatus is double. I find it has the resemblance of two teeth ossified. It is very firmly set. About two years ago he had a city dentist to examine it. The dentist told him that the tooth was decayed internally; he drilled a hole into it, and found nothing but a thick shell. It does not seem to decay very rapidly, and may last several years. He "won't" have it filled.

I give the above without comment. The first is the most singular, and may be doubted by some. The balance are more common. At some future day, I intend to gather all the facts of the first case in this paper, and give them to the profession, together with such idiosyncracies as the case may present.

